# Addition of immunotherapy to perioperative chemotherapy for resectable gastric and gastroesophageal junction cancer: a meta-analysis of phase 2/3 trials

**DOI:** 10.3389/fimmu.2025.1692336

**Published:** 2025-11-19

**Authors:** Yuxuan Lin, Yonghe Liao, Jinhai Shen

**Affiliations:** 1 Department of Pharmacy, Guangxi Hospital Division of The First Affiliated Hospital, Sun Yat-sen University, Nanning, Guangxi, China; 2 School of Pharmaceutical Science, Guangxi Medical University, Nanning, Guangxi, China; 3 State Key Laboratory of Natural Medicines, China Pharmaceutical University, Nanjing, Jiangsu, China; 4 Center for New Drug Safety Evaluation and Research, China Pharmaceutical University, Nanjing, Jiangsu, China; 5 School of Basic Medicine and Clinical Pharmacy, China Pharmaceutical University, Nanjing, Jiangsu, China

**Keywords:** immune checkpoint inhibitors, chemotherapy, gastric cancer, perioperative treatment, meta-analysis

## Abstract

**Background:**

The integration of immune checkpoint inhibitors (ICIs) with perioperative chemotherapy (CT) has become a major focus of clinical research in resectable gastric or gastroesophageal junction (G/GEJ) cancer. Recent phase 2 and 3 trials have reported disparate outcomes, generating considerable debate. To synthesize this evidence, we conducted a meta-analysis to evaluate the efficacy and safety of adding ICIs to CT in this setting.

**Methods:**

A systematic literature search of PubMed and major conference proceedings was conducted up to August 15, 2025. Efficacy outcomes included summary relative risks (RRs) for pathological complete response (pCR) and R0 resection rate, and hazard ratios (HRs) for event-free survival (EFS) and overall survival (OS). Safety was assessed using RRs for treatment-related adverse events (AEs).

**Results:**

Four trials (one phase 2 [NEOSUMMIT-01], one phase 2/3 [DANTE], and two phase 3 [KEYNOTE-585, MATTERHORN]), encompassing 2,358 patients, were included. The addition of ICIs to CT significantly improved pCR (RR: 2.80; 95% CI: 1.68–4.67), EFS (HR: 0.76; 95% CI: 0.67–0.87), and OS (HR: 0.78; 95% CI: 0.61–0.99), although it did not increase the R0 resection rate. Regarding safety, the combination did not increase the risk of overall grade 3–5 treatment-related AEs. However, it was associated with a significantly higher risk of grade 3–5 immune-related AEs (RR: 2.88; 95% CI: 1.95–4.24) and treatment-related serious AEs (RR: 1.14; 95% CI: 1.01–1.28).

**Conclusion:**

The addition of ICIs to perioperative CT confers significant improvements in pCR, EFS, and OS in resectable G/GEJ cancer, with a generally manageable safety profile. However, longer follow-up is required to validate survival benefits, and the increased risk of immune-related toxicity underscores the need for administration in specialized centers. These findings suggest that perioperative chemo-immunotherapy is a promising treatment strategy, though its definitive role awaits confirmation from ongoing phase 3 trials.

**Systematic review registration:**

https://www.crd.york.ac.uk/prospero/, identifier CRD420251131385.

## Introduction

Gastric and gastroesophageal junction (G/GEJ) cancers represent a major global health burden, ranking among the most common and lethal malignancies worldwide (1, 2). Despite advances in surgical techniques and perioperative management, prognosis for patients with locally advanced disease remains poor, with recurrence occurring in a substantial proportion of cases even after curative resection (3). Multimodality strategies, particularly the incorporation of perioperative chemotherapy (CT), have been established as the cornerstone of therapy to improve survival (4, 5). Landmark studies such as MAGIC and FLOT4 demonstrated that perioperative regimens enhance the probability of achieving R0 resection and improve overall survival (OS) compared with surgery alone, thereby shaping current treatment standards (4, 6). Nevertheless, long-term outcomes remain unsatisfactory, with 5-year survival rates rarely exceeding 50%, underscoring the need for novel therapeutic approaches.

In recent years, immune checkpoint inhibitors (ICIs) targeting programmed death 1 or its ligand 1 (PD-1/PD-L1) have revolutionized the treatment landscape of advanced and metastatic G/GEJ cancers (7). Large phase 3 trials including CheckMate 649, KEYNOTE-859, and GEMSTONE-303 established PD-1/PD-L1 blockade in combination with CT as a new standard for metastatic disease, demonstrating clinically meaningful improvements in OS (8–10). This success has stimulated interest in moving ICIs into earlier stages of disease, with the hope of inducing deeper pathological responses, eradicating micrometastatic disease, and ultimately improving cure rates when combined with perioperative CT.

Several clinical trials have now evaluated this strategy in the resectable setting. The randomized phase 2 NEOSUMMIT-01 trial reported that adding the PD-1 inhibitor toripalimab to perioperative SOX/XELOX CT significantly increased pathological complete or near-complete response rates compared with CT alone, with manageable safety (11). Similarly, the German DANTE trial demonstrated that atezolizumab combined with perioperative FLOT improved histopathological regression and tumor downstaging, particularly in biomarker-defined subgroups, without excess surgical morbidity (12). More definitive evidence emerged from phase 3 studies. The global KEYNOTE-585 trial tested pembrolizumab plus perioperative CT and showed significant improvements in pathological complete response (pCR), though event-free survival (EFS) benefits did not reach statistical significance (13, 14). In contrast, the multinational MATTERHORN trial demonstrated that perioperative durvalumab plus FLOT significantly improved EFS compared with CT alone, establishing proof of concept in a phase 3 setting (15).

Taken together, these trials provide important, albeit heterogeneous, evidence supporting the integration of ICIs with perioperative CT for resectable G/GEJ cancers. However, the magnitude of clinical benefit and its consistency across different ICI agents, CT backbones, and patient subgroups remain subjects of active investigation. To address this knowledge gap, we conducted a systematic review and meta-analysis of phase 2 and 3 randomized trials to evaluate the efficacy and safety of adding ICIs to perioperative CT in patients with resectable G/GEJ cancers.

## Methods

### Protocol and reporting guidelines

The study was prospectively registered in (CRD420251131385) and conducted in accordance with the PRISMA 2020 reporting guidelines (16).

### Information sources and search strategy

A comprehensive literature search of PubMed was performed to capture all eligible phase 2 and 3 clinical trials available up to August 15, 2025. Search terms included both Medical Subject Headings and free-text keywords, with the full strategy provided in **Supplementary Table 1**. Abstracts from major oncology congresses (e.g., ASCO, ESMO) were also reviewed to supplement the database search and identify relevant grey literature.

### Selection criteria

Eligible studies were required to satisfy the following conditions: (i) randomized phase 2 or 3 trials directly comparing ICI-combined CT regimens against CT alone; (ii) enrollment of patients with resectable G/GEJ cancer; and (iii) availability of key outcomes data. Exclusion criteria comprised: (i) studies not designed as phase 2/3 randomized controlled trials (RCTs); (ii) absence of a CT-only control arm; (iii) omission of ICIs in the investigational arm; (iv) interventions limited to either the neoadjuvant or adjuvant setting rather than both; and (v) trials still ongoing without publicly available results at the time of the search. Only studies that fulfilled these predefined eligibility criteria were incorporated into the meta-analysis.

### Data collection and assessment of risk of bias

Data extraction was carried out by one investigator and independently validated by a second reviewer. Information collected included trial name, year of publication, sample size, principal eligibility criteria, treatment regimens, pCR rates, and hazard ratios (HRs) with corresponding 95% confidence intervals (CIs) for EFS and overall survival (OS). Safety outcomes were also recorded, specifically the frequency of grade 3–5 treatment-related adverse events (AEs), grade 3–5 immune-related AEs, and serious AEs. In addition, study design characteristics were retrieved to allow assessment of potential bias, which was performed using the Cochrane risk-of-bias tool (17).

### Statistical analysis

Pooled effect estimates were generated using either fixed- or random-effects models, depending on the degree of heterogeneity. Statistical heterogeneity was quantified with the *I²* statistic and assessed by the Cochrane *Q* test, with significance defined as *I²* >50% and a *Q* test *p* < 0.10. Both statistical and clinical heterogeneity informed model selection, with random-effects models applied when substantial variability was anticipated, and fixed-effects models employed when homogeneity was well supported.

For efficacy outcomes, relative risks (RRs) with 95% CIs were calculated for pCR and R0 resection, and HRs with 95% CIs for EFS and OS. For safety endpoints, RRs with 95% CIs were pooled for AE outcomes. Random-effects models were consistently used in subgroup analyses due to limited power to exclude heterogeneity. Publication bias was evaluated through funnel plots and Egger’s regression test. All statistical analyses were performed in R (version 4.5.1), with two-sided *p* < 0.05 considered significant.

## Results

### Study selection and characteristics of included studies

A total of 94 records were retrieved through the literature search. After screening and eligibility assessment, four trials fulfilled the inclusion criteria and were incorporated into the final analysis (11–15). The PRISMA flow chart for the study selection process is provided in **Supplementary Figure 1**.

Of the four eligible trials, one was an open-label phase 2 RCT (11), one an open-label phase 2/3 RCT (12), and two were double-blind phase 3 RCTs (13–15). Collectively, these studies enrolled 2,358 patients with resectable G/GEJ cancer, including 1,176 (49.9%) treated with ICIs plus CT and 1,182 (50.1%) treated with CT alone. The immunotherapy agents tested were toripalimab and pembrolizumab (PD-1 inhibitors) and atezolizumab and durvalumab (PD-L1 inhibitors). Control regimens consisted of SOX (S-1 plus oxaliplatin), XELOX (capecitabine plus oxaliplatin), FLOT (fluorouracil, leucovorin, oxaliplatin, and docetaxel), and cisplatin-based chemotherapy. An overview of trial characteristics is provided in **Table 1**.

**Table 1:** Characteristics of the included trials.

Study	Year	Design	Key eligible criteria	Sample size (exp/ctrl)	Drugs used in treatment		Treatment regimens	pCR	mEFS HR (95% CI)	mOS HR (95% CI)
					Exp arm	Ctrl arm				
NEOSUMMIT-01 (NCT04250948)	2024	Phase II, open-label RCT	Histologically confirmed cT3-4aN+M0 G/GEJ adenocarcinoma, age 18-75, ECOG PS 0-1, no contraindications for surgery, M0 confirmed by diagnostic laparoscopy.	108 54/54	Toripalimab plus SOX/XELOX	SOX / XELOX	Exp arm: 3 preoperative plus 5 postoperative cycles of SOX/XELOX plus toripalimab, followed by ≤6 moths of toripalimab maintenance Ctrl arm: 3 preoperative plus 5 postoperative cycles of SOX/XELOX	22.2% vs. 7.4%	NA	NA
DANTE (NCT03421288)	2024	Phase II/III, open-label RCT	Resectable G/GEJ adenocarcinoma, clinical stage ≥cT2 and/or cN+, M0 confirmed by endoscopy/imaging, age ≥18, ECOG PS 0-1	295 (146/149)	Atezolizumab plus FLOT	FLOT	Exp arm: 4 preoperative plus 4 postoperative cycles of FLOT plus atezolizumab, followed by 8 cycles of atezolizumab maintenance Ctrl arm: 4 preoperative plus 4 postoperative cycles of FLOT	24.0% *vs.* 14.8%	NA	NA
KEYNOTE-585 (NCT03221426)	2024	Phase III, placebo-controlled, double-blind RCT	Untreated locally advanced resectable G/GEJ adenocarcinoma (T3+ or N+ per AJCC), age ≥18, ECOG PS 0-1, life expectancy ≥6 months	804 (402/402)	Pembrolizumab plus cisplatin-based CT	Placebo plus cisplatin-based CT	Exp arm: Neoadjuvant (3 cycles) plus adjuvant (3 cycles) pembrolizumab plus cisplatin-based CT, followed by 11 cycles of pembrolizumab maintenance Ctrl arm: Neoadjuvant (3 cycles) plus adjuvant (3 cycles) placebo plus cisplatin-based CT, followed by 11 cycles of placebo	14.2% *vs.* 2.8%	47.0 *vs.* 26.9 0.80 (0.67-0.95)	NR vs. 55.7 0.86 (0.71-1.03)
				203 (100/103)	Pembrolizumab plus FLOT	Placebo plus FLOT	Exp arm: 4 preoperative plus 4 postoperative cycles of FLOT plus pembrolizumab, followed by 11 cycles of pembrolizumab maintenance Ctrl arm: 4 preoperative plus 4 postoperative cycles of FLOT plus placebo, followed by 11 cycles of placebo			
MATTERHORN (NCT04592913)	2025	Phase III, placebo-controlled, double-blind RCT	Age ≥18 year; histologically confirmed resectable G/GEJ adenocarcinoma (stage II-IVA); ECOG PS 0-1; adequate organ function; tumor sample for PD-L1 testing; no prior anticancer therapy	948 (474/474)	Durvalumab plus FLOT	Placebo plus FLOT	Exp arm: 2 preoperative plus 2 postoperative cycles of FLOT plus durvalumab (q4w), followed by 10 cycles of durvalumab maintenance Ctrl arm: 2 preoperative plus 2 postoperative cycles of FLOT plus placebo (q4w), followed by 10 cycles of placebo	19.2% *vs.* 7.2%	NR *vs.* 32.8 0.71 (0.58-0.86)	NR *vs.* NR 0.67 (0.50-0.90)

RCT, randomized controlled trial; ECOG PS, Eastern Cooperative Oncology Group performance status; Exp, experimental; Ctrl, control; SOX, S-1 plus oxaliplatin; XELOX, capecitabine plus oxaliplatin; FLOT, fluorouracil, leucovorin, oxaliplatin, docetaxel; CT, chemotherapy; pCR, pathological complete response; mEFS, median event-free survival; mOS, median overall survival; HR, hazard ratio; CI, confidence interval; NA, not available; NR: not reached.

### Efficacy in intention-to-treat population

**Figure 1:**
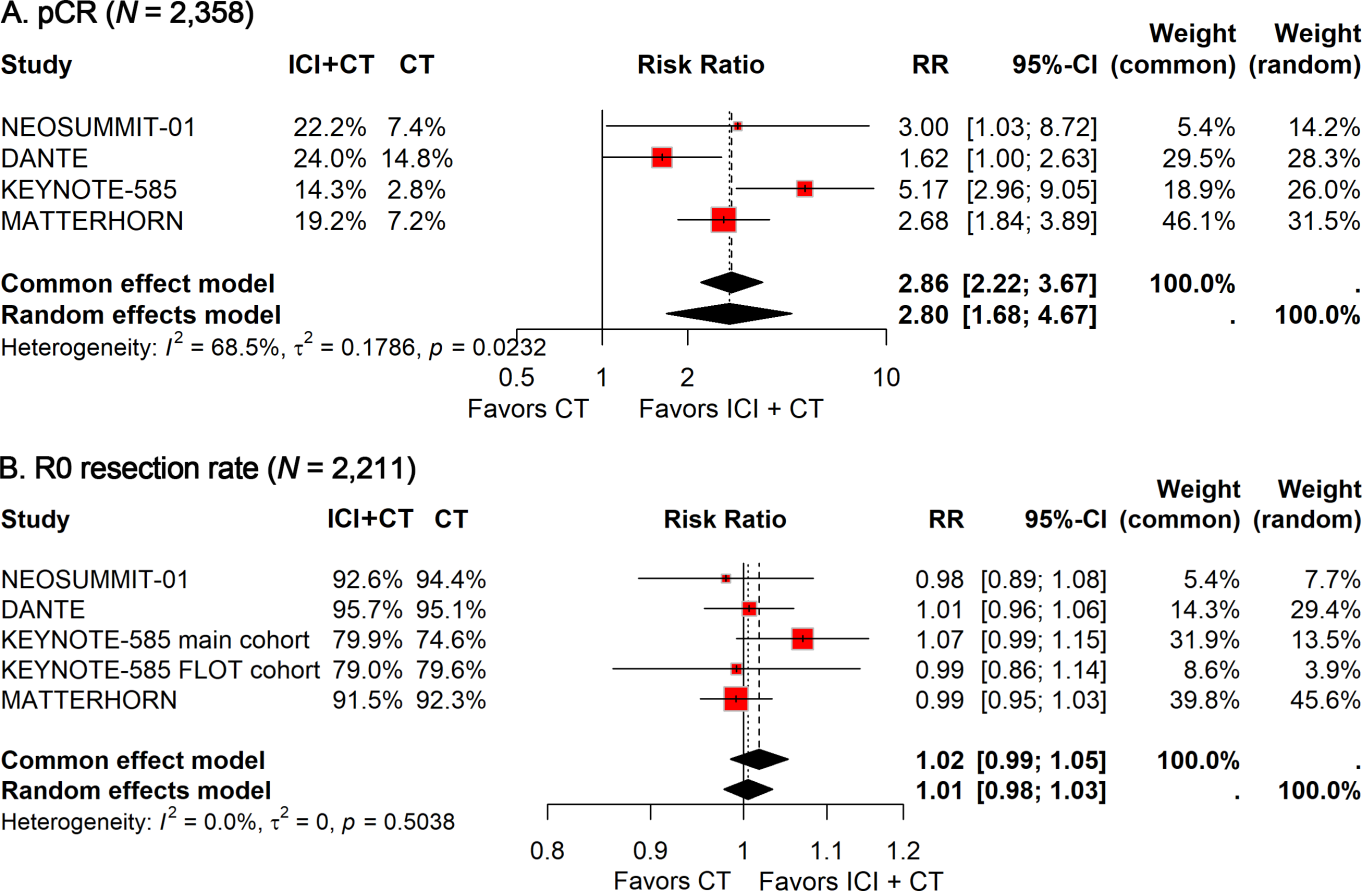
Forest plot of pCR **(A)** and R0 resection rate **(B)** comparing ICIs plus CT *versus* CT alone in patients with resectable G/GEJ cancer.

All the included studies reported the pCR and R0 resection rates. Given the significant heterogeneity observed (*I²* = 68.5%), the random-effects model was adopted to calculate the pooled RR. Meta-analysis indicated that the addition of ICIs to perioperative CT significantly improved pCR (RR: 2.80; 95% CI: 1.68–4.67; **Figure 1A**). However, no significant difference was observed in the R0 resection rate (RR: 1.02; 95% CI: 0.99–1.05; **Figure 1B**), with no heterogeneity detected (*I²* = 0).

**Figure 2:**
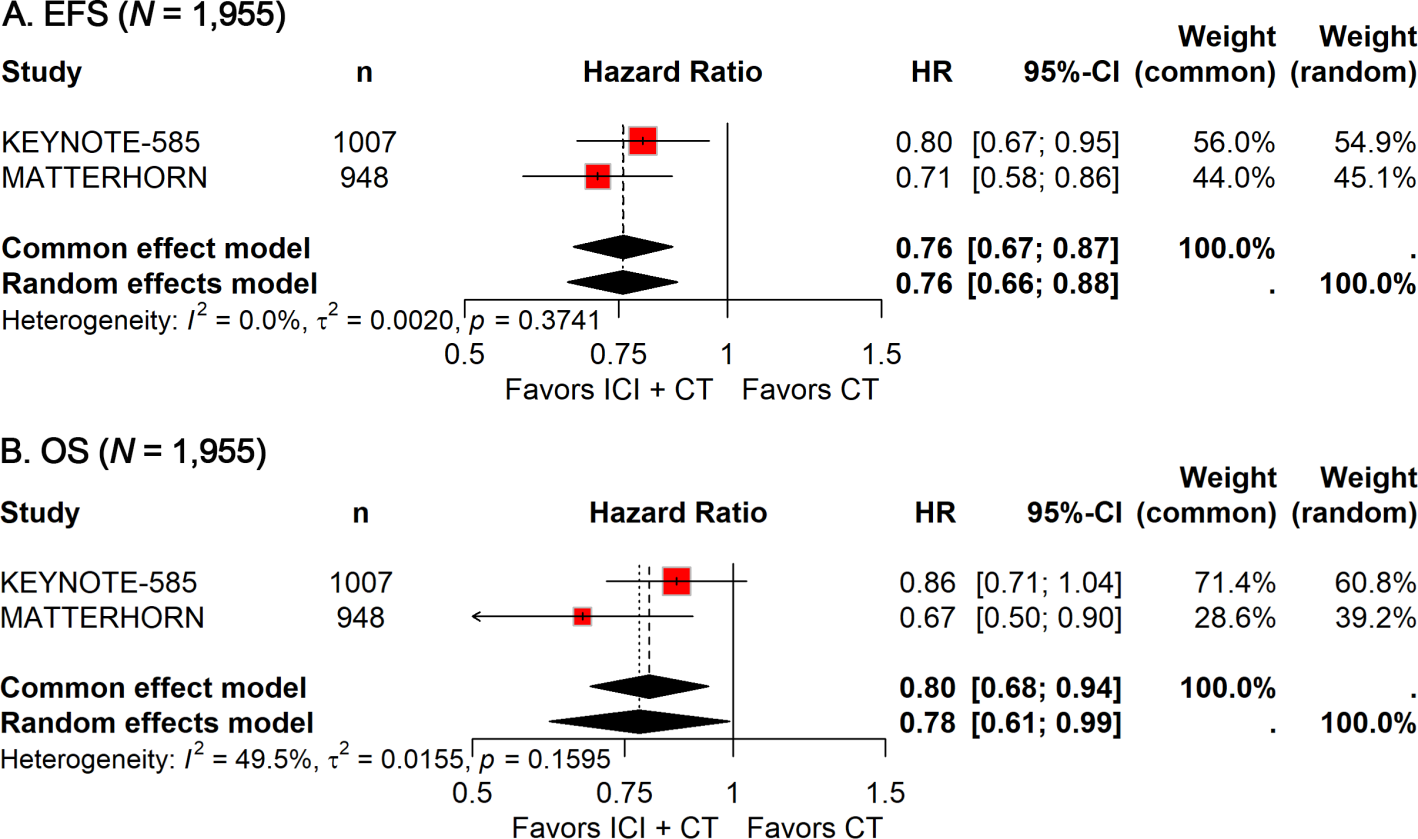
Forest plot of EFS **(A)** and OS **(B)** comparing ICIs plus CT *versus* CT alone in patients with resectable G/GEJ cancer.

EFS and OS data were available from two phase 3 trials (13–15), encompassing a total of 1,955 patients. The pooled HR for EFS demonstrated a significant benefit with ICI-CT compared with CT alone (HR: 0.76; 95% CI, 0.67–0.87; **Figure 2A**), corresponding to a 24% relative reduction in the risk of events. Notably, no heterogeneity was observed (*I²* = 0). For OS, the combined HR indicated a 22% reduction in the risk of death with ICI-CT (HR: 0.78; 95% CI: 0.61–0.99; **Figure 2B**), with moderate heterogeneity observed (*I²* = 49.5%). Collectively, these results demonstrate consistent and substantial efficacy of ICI-CT regimens across survival endpoints.

### Safety in ITT population

**Figure 3:**
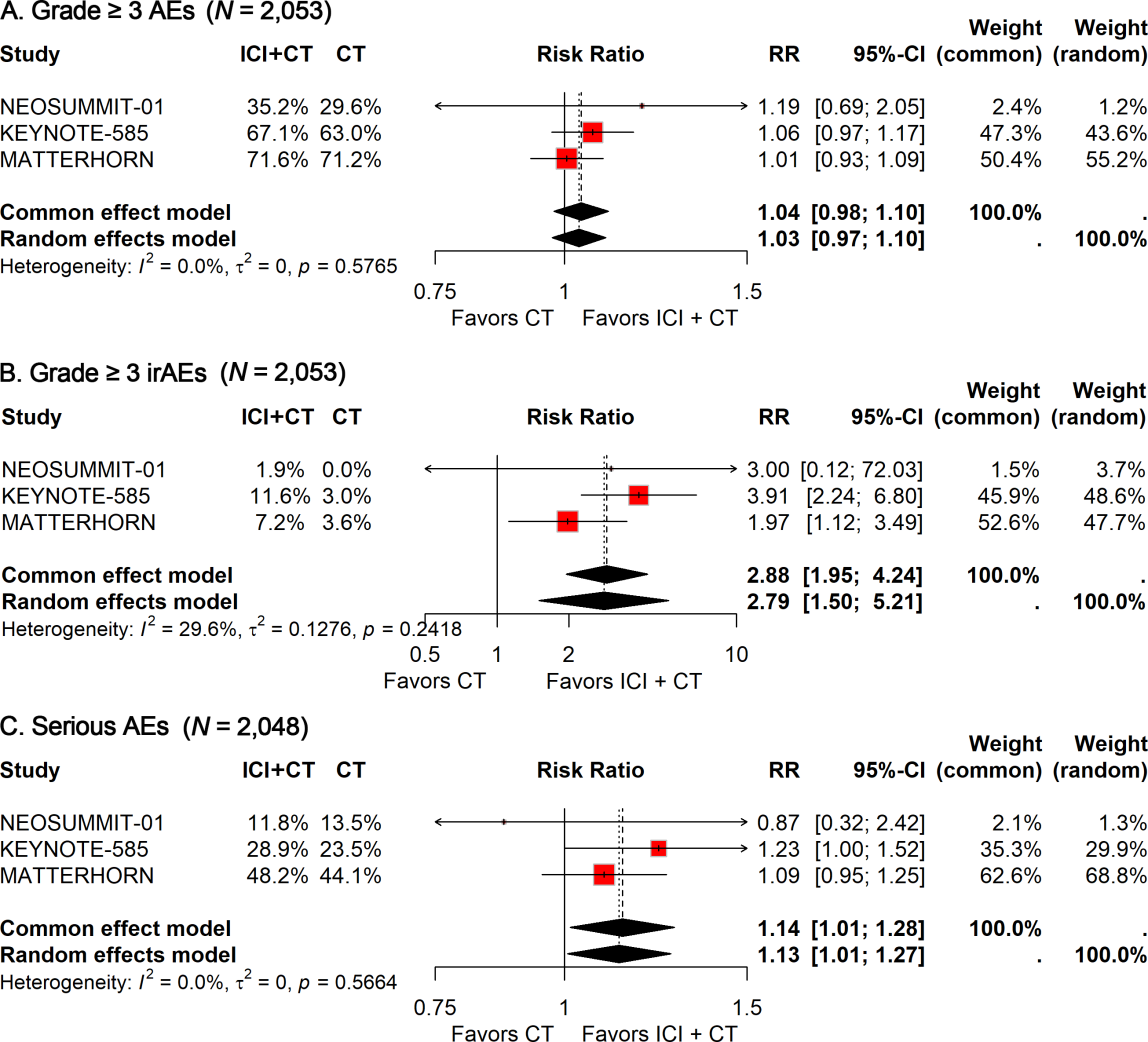
Forest plot of grade 3–5 treatment-related AEs **(A)**, grade 3–5 immune-related AEs **(B)**, and treatment-related serious AEs **(C)** comparing ICIs plus CT *versus* CT alone in patients with resectable G/GEJ cancer stratified by age.

Among the 1,027 patients treated with ICIs plus CT, 693 (67.5%) experienced grade 3–5 treatment-related AEs, compared with 667 of 1,026 patients (65.0%) receiving CT alone. With no heterogeneity detected (*I²* = 0%), a fixed-effects model was applied to calculate the pooled RR. The analysis showed that the addition of ICIs did not significantly increase the incidence of grade 3–5 treatment-related AEs (RR, 1.04; 95% CI, 0.98-1.10; **Figure 3A**).

For grade 3–5 immune-related AEs, 93 of 1,027 patients (9.1%) in the ICI–CT group were affected, compared with 32 of 1,026 patients (3.1%) in the CT group. Given the low heterogeneity observed (*I²* = 29.6%), a fixed-effects model was again used. The pooled analysis demonstrated a significantly higher risk of grade 3–5 immune-related AEs with the addition of ICIs (RR: 2.88; 95% CI: 1.95–4.24; **Figure 3B**).

Safety data on serious AEs were available from three trials. The incidence of serious AEs was 37.0% (379/1,024) in the ICI–CT group versus 32.4% (332/1,024) in the CT group. Pooled estimates indicated a significantly greater risk of serious AEs in patients receiving ICI–CT compared with CT alone (RR: 1.14; 95% CI: 1.01–1.28; **Figure 3C**).

**Figure 4:**
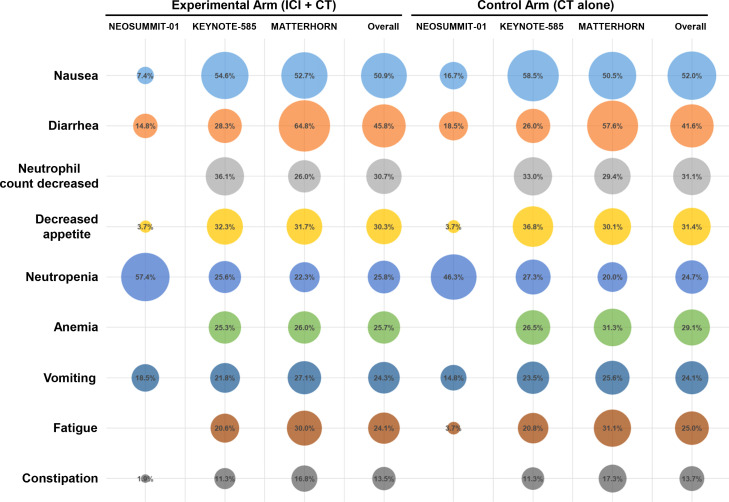
Matrix bubble plot summarizing the most prevalent treatment-related AEs of any grade.

**Figure 5:**
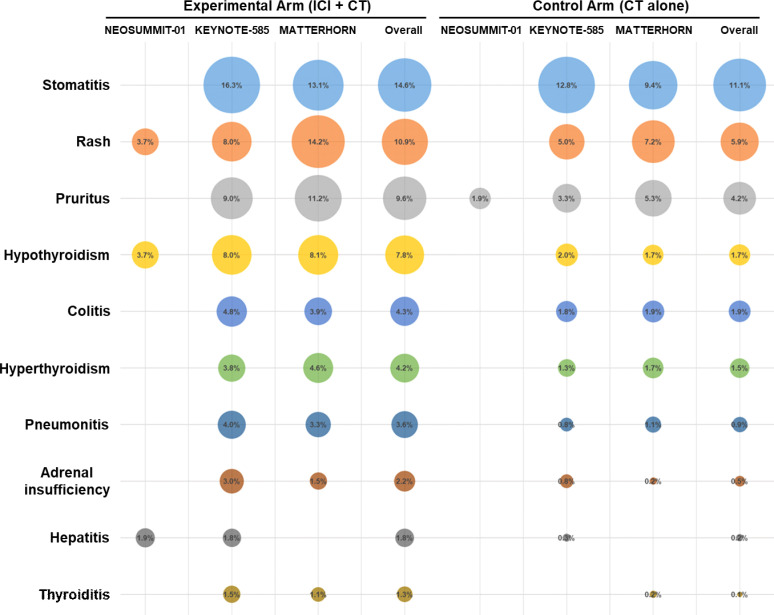
Matrix bubble plot summarizing the most prevalent immune-related AEs of any grade.

A detailed evaluation was conducted to determine the incidence of all-grade treatment-related AEs reported across the included trials. The most commonly observed events were nausea (50.9% with ICI plus CT vs. 52.0% with CT alone), diarrhea (45.8% vs. 41.6%), and neutrophil count decreased (30.7% vs. 31.1%), followed by decreased appetite and neutropenia (**Figure 4**). The overall distribution of these frequent treatment-related AEs was largely comparable between the two treatment groups. In addition, among patients receiving ICI–CT regimens, the most frequent immune-related toxicities included stomatitis (14.6%), rash (10.9%), pruritus (9.6%), hypothyroidism (7.8%), and colitis (4.3%) (**Figure 5**).

### Subgroup analysis

Given the limited statistical power of individual trials to evaluate clinically relevant subgroups, we performed a series of subgroup analyses to further characterize the efficacy of ICIs combined with CT in defined patient populations and to provide insights for individualized precision treatment.

#### Subgroup analysis stratified by patient demographics

To assess the influence of demographic factors on treatment efficacy, subgroup analyses were conducted according to age, sex, geographic region, and Eastern Cooperative Oncology Group (ECOG) performance status.

**Figure 6:**
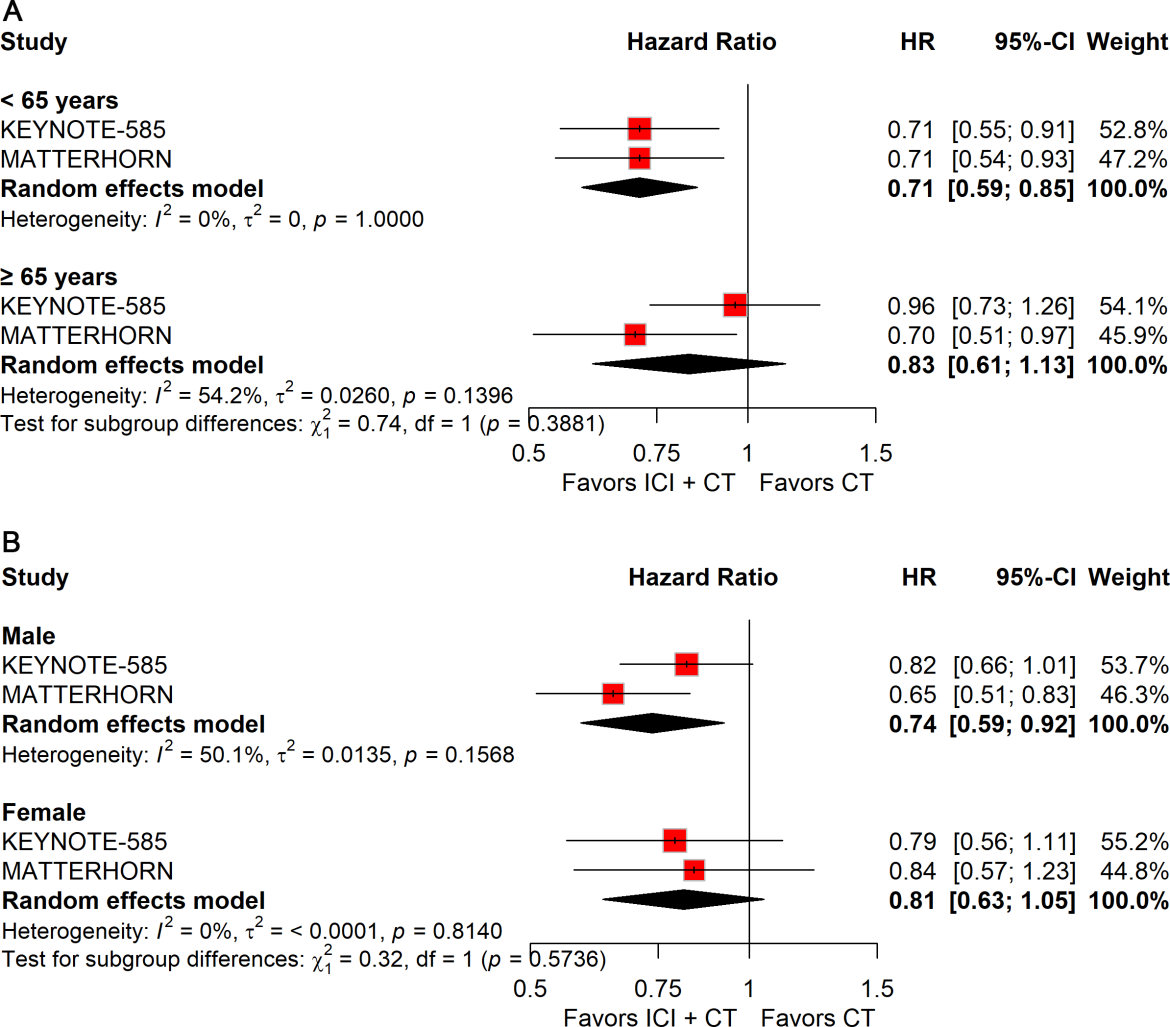
Forest plot of EFS comparing ICIs plus CT *versus* CT alone in patients with resectable G/GEJ cancer stratified by age **(A)** and sex **(B)**.

Analysis by age demonstrated that the addition of ICIs to CT significantly prolonged EFS in patients younger than 65 years (HR: 0.71; 95% CI: 0.59–0.85), but not in those aged ≥65 years (**Figure 6A**).

Stratification by sex revealed a significant EFS benefit in male patients (HR: 0.74; 95% CI: 0.59–0.92), whereas no survival improvement was observed in female patients (**Figure 6B**).

**Figure 7:**
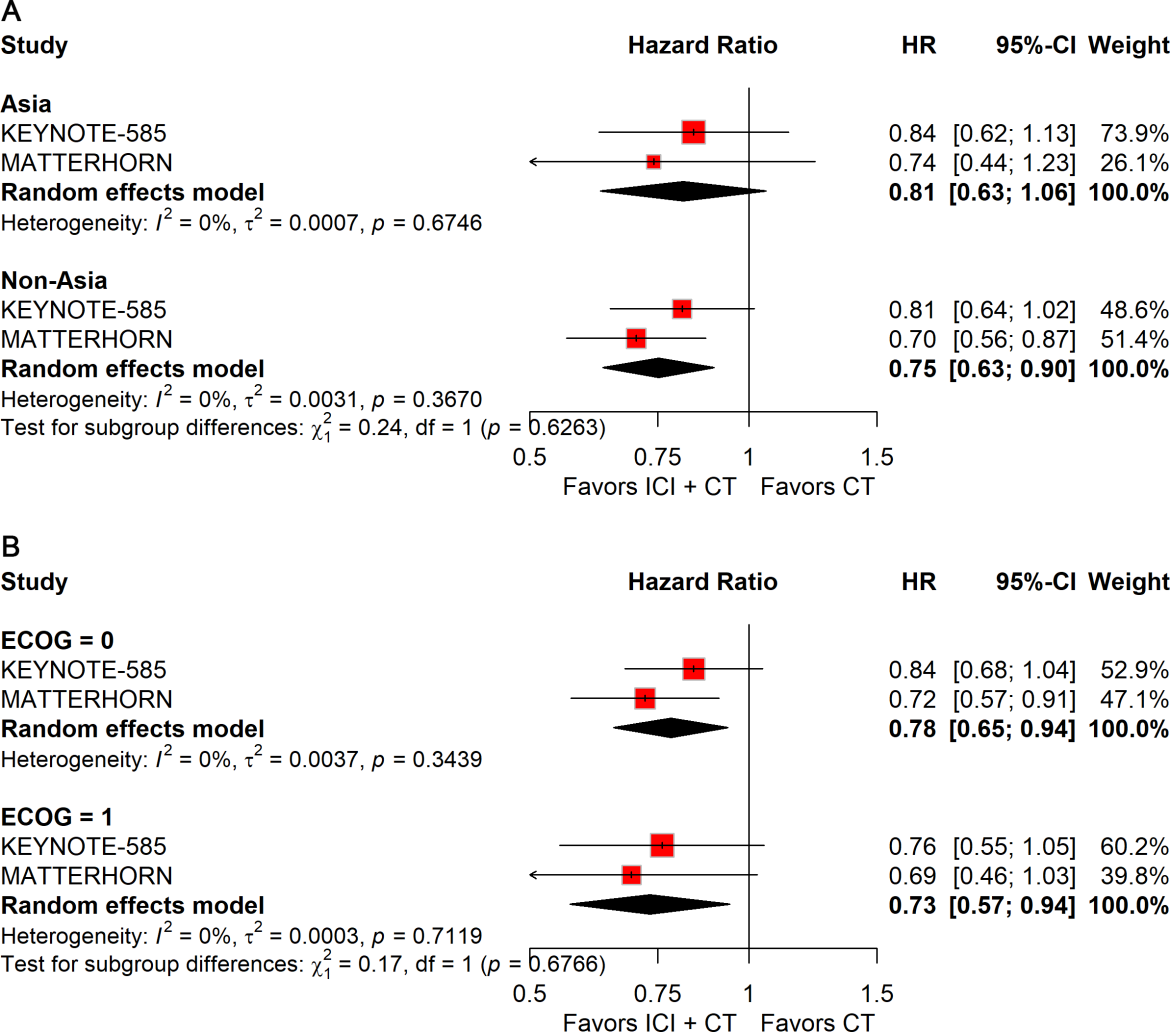
Forest plot of EFS comparing ICIs plus CT *versus* CT alone in patients with resectable G/GEJ cancer stratified by region **(A)** and ECOG performance status **(B)**.

Regional stratification showed a significant benefit for patients enrolled outside Asia (HR: 0.75; 95% CI: 0.63–0.90), but not for those from Asia (**Figure 7A**).

Analyses by ECOG status indicated consistent improvements in EFS across subgroups: ECOG 0 (HR: 0.78; 95% CI: 0.65–0.94) and ECOG 1 (HR: 0.73; 95% CI: 0.57–0.94) (**Figure 7B**).

#### Subgroup analysis stratified by disease pathology

To evaluate whether disease-related characteristics influenced outcomes, subgroup analyses were conducted according to PD-L1 expression, mismatch repair (MMR)/microsatellite instability (MSI) status, primary tumor site, and histologic subtype.

**Figure 8:**
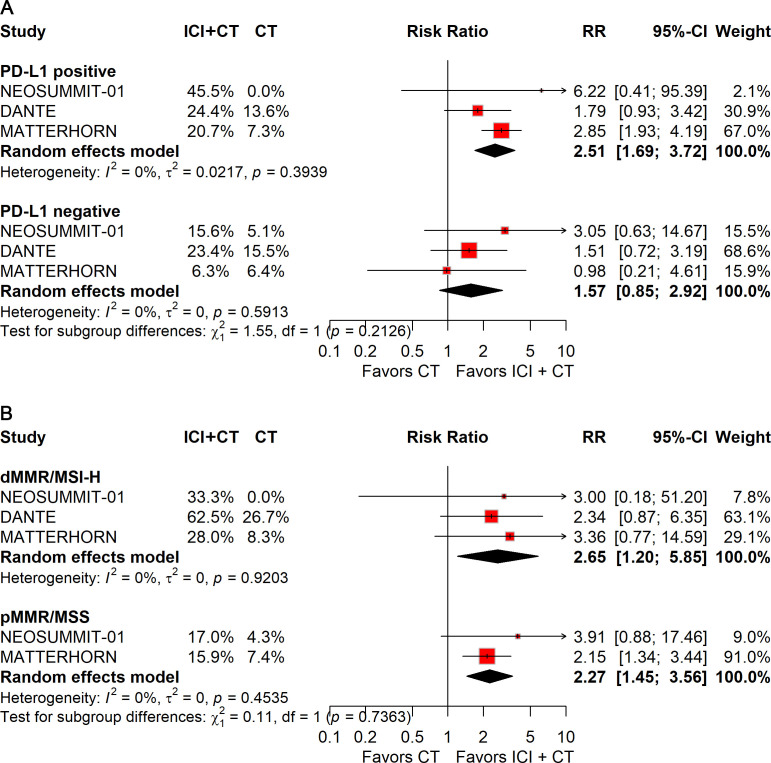
Forest plot of pCR comparing ICIs plus CT *versus* CT alone in patients with resectable G/GEJ cancer stratified by PD-L1 expression **(A)** and MMR/MSI status **(B)**.

**Figure 9:**
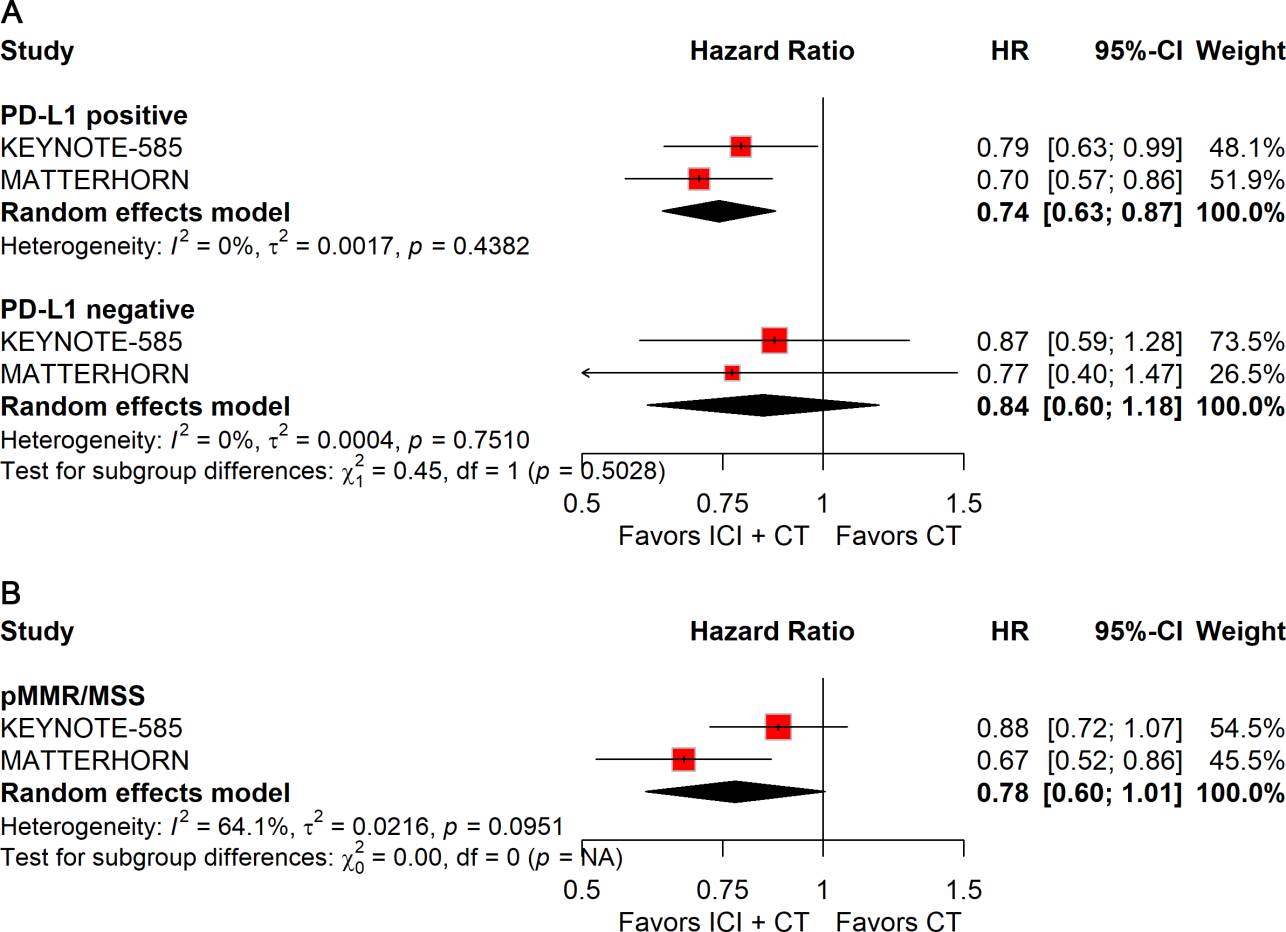
Forest plot of EFS comparing ICIs plus CT *versus* CT alone in patients with resectable G/GEJ cancer stratified by PD-L1 expression **(A)** and MMR/MSI status **(B)**.

Patients with PD-L1–positive tumors derived significant improvements in both pCR (RR: 2.51; 95% CI: 1.69–3.72; **Figure 8A**) and EFS (HR: 0.74; 95% CI: 0.63–0.87; **Figure 9A**), whereas no benefit was observed in PD-L1–negative patients.

Stratification by MMR/MSI status revealed significant gains in pCR with ICIs plus CT for both dMMR/MSI-H (RR: 2.65; 95% CI: 1.20–5.85) and pMMR/MSS (RR: 2.27; 95% CI: 1.45–3.56) tumors (**Figure 8B**). However, patients with pMMR/MSS showed no significant improvement in EFS; data for pMMR/MSI-H were unavailable (**Figure 9B**).

**Figure 10:**
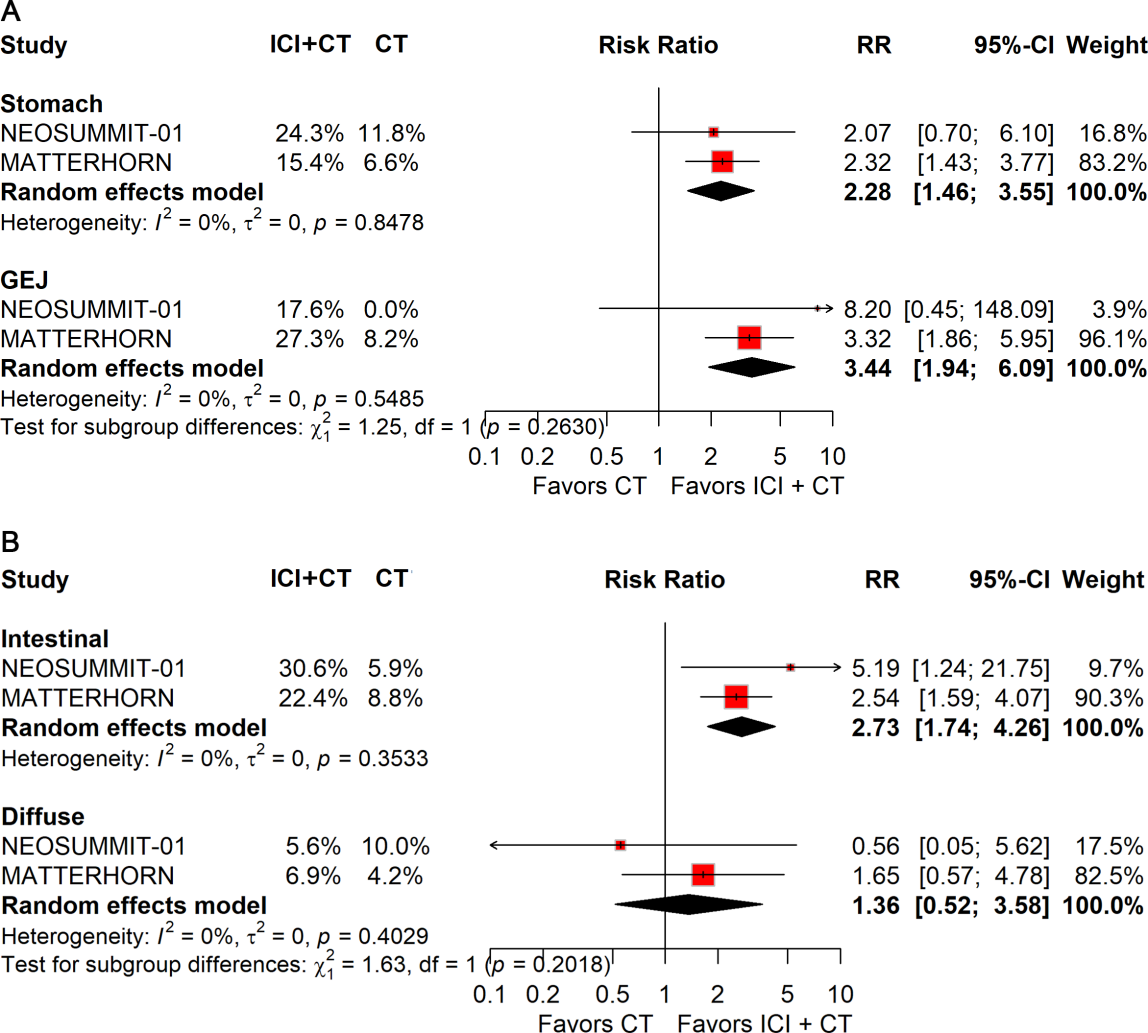
Forest plot of pCR comparing ICIs plus CT *versus* CT alone in patients with resectable G/GEJ cancer stratified by primary tumor location **(A)** and pathological type **(B)**.

**Figure 11:**
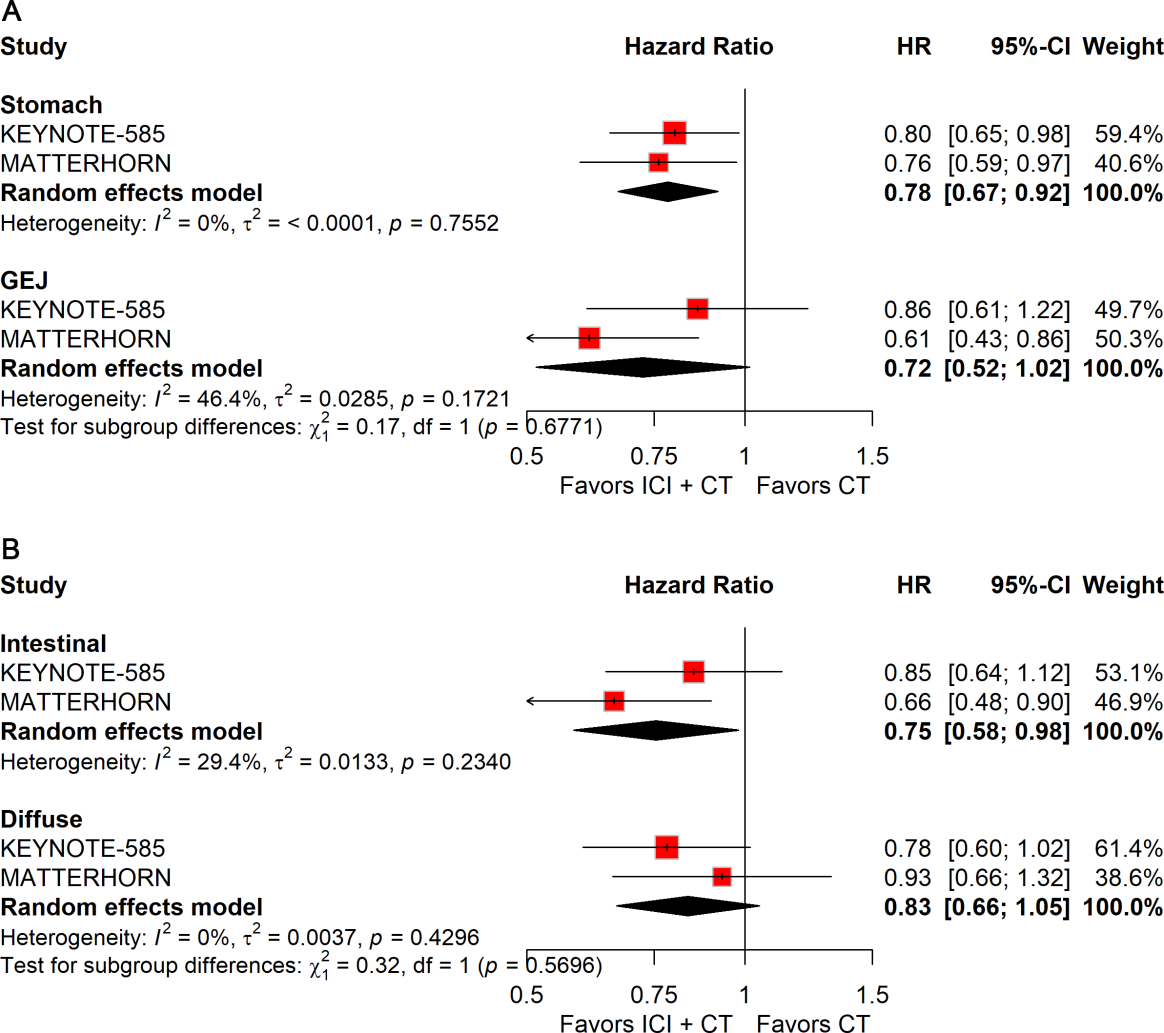
Forest plot of EFS comparing ICIs plus CT *versus* CT alone in patients with resectable G/GEJ cancers stratified by primary tumor location **(A)** and pathological type **(B)**.

When stratified by primary tumor location, significant improvements in pCR were observed in both gastric (RR: 2.28; 95% CI: 1.46–3.55) and gastroesophageal junction (GEJ) cancers (RR: 3.44; 95% CI: 1.94–6.09; **Figure 10A**). In contrast, for EFS, only gastric cancer (HR: 0.78; 95% CI: 0.67–0.92; **Figure 11A**) demonstrated a significant benefit, whereas GEJ cancer did not.

Histological stratification showed that patients with intestinal-type tumors derived consistent benefits in both pCR (RR: 2.73; 95% CI: 1.74–4.26; **Figure 10B**) and EFS (HR: 0.75; 95% CI: 0.58–0.98; **Figure 11B**). By comparison, no significant differences were observed in patients with diffuse-type tumors.

### Risk of bias

The risk-of-bias assessment for the included trials is illustrated in **Supplementary Figures 2**, **3**. Both the NEOSUMMIT-01 and DANTE studies were randomized, open-label trials with investigator-assessed outcomes and pre-specified analytical strategies. Accordingly, they were judged to carry a low risk of selection, detection, attrition, and reporting bias, but a high risk of performance bias attributable to the open-label design. In contrast, the two randomized, placebo-controlled, double-blind phase 3 trials were considered to have a low risk of bias across all evaluated domains. Funnel plot inspection and Egger’s regression test revealed no indication of publication bias (**Supplementary Figure 4**).

## Discussion

This meta-analysis synthesizing data from four randomized phase 2/3 trials (NEOSUMMIT-01, DANTE, KEYNOTE-585, and MATTERHORN) including 2,358 patients with resectable G/GEJ cancer provides the most comprehensive evaluation to date of adding ICIs to perioperative CT. The pooled results demonstrate consistent and clinically meaningful improvements in pCR, EFS, and OS, achieved without compromising R0 resection rates or surgical feasibility. These findings establish perioperative chemo-immunotherapy as a transformative new standard of care for locally advanced, resectable G/GEJ cancers.

While the efficacy of ICIs in metastatic gastric cancer is established, their role in the perioperative setting—encompassing both neoadjuvant and adjuvant phases—is still being defined. Although several meta-analyses have explored ICIs in resectable G/GEJ cancer, a dedicated synthesis of phase 2/3 RCTs for a strictly perioperative ICI-CT strategy (administered both pre- and post-surgery) is lacking. Previous syntheses have included a mix of neoadjuvant-only, adjuvant-only, and perioperative regimens, leaving this specific niche unfilled (18–20). Our findings consolidate and extend the evidence from a previously published meta-analysis by Huang et al., which demonstrated a significant improvement in pCR but was limited to short-term pathological and safety outcomes (21). Our study provides several critical advancements. First, by strictly focusing on ICI-CT combinations and excluding trials incorporating additional targeted therapies (e.g., anti-angiogenic agents), we offer a more precise estimate of the effect attributable to immunotherapy alongside CT. Second, and most importantly, our analysis incorporates key survival endpoints, providing the first meta-analytic evidence of a significant improvement in EFS and a clinically meaningful reduction in the risk of death (OS). Finally, our extensive subgroup analyses deliver unprecedented insights into the differential efficacy across key patient subgroups defined by PD-L1 status, MMR/MSI, histology, and region, thereby moving the field beyond the simple question of ‘if’ the combination works toward understanding ‘for whom’ it works best.

A cornerstone of this analysis is the nearly threefold increase in pCR rate with ICI combination therapy. This benefit is consistent across trials: NEOSUMMIT-01 (toripalimab + SOX/XELOX) reported a pCR rate of 22.2% vs. 7.4% with CT alone; MATTERHORN (durvalumab + FLOT) showed 19.2% vs. 7.2%; even KEYNOTE-585 (pembrolizumab + cisplatin-based CT)—which missed its pre-specified EFS threshold (HR = 0.81, *p* = 0.0198 vs. α = 0.0178)—still demonstrated a 14.2% vs. 2.8% pCR advantage. This builds on the legacy of perioperative CT, but ICIs nearly double or triple this rate, reflecting synergistic antitumor activity.

Survival outcomes reinforce the clinical promise of perioperative immunotherapy, with a 24% relative reduction in progression or death (EFS HR 0.76) and 22% reduction in mortality (OS HR 0.78). The consistent benefit across PD-1 and PD-L1 inhibitors supports a class effect, while differences between MATTERHORN (positive) and KEYNOTE-585 (borderline) underscore the influence of trial design, patient selection, and CT platform. Parallels to other solid tumors where early ICIs integration has altered treatment landscapes exemplify the shifting paradigm toward immunotherapy-enabled curative approaches in G/GEJ cancer (22–24).

Subgroup analyses reveal meaningful heterogeneity relevant to personalized treatment. PD-L1 positivity strongly predicts benefit in both pCR and EFS, solidifying its role as the most reliable biomarker currently available. Patients with dMMR/MSI-H tumors achieve substantial pCR improvements, aligning with biological rationale for immunotherapy sensitivity. Even pMMR/MSS tumors exhibit pCR enhancements, albeit with less certain survival gains, suggesting transient CT-induced immune priming. Histological and geographic disparities emerge: intestinal-type and non-Asian patients experience clear benefits, whereas diffuse-type tumors and Asian cohorts show limited responses. These nuances highlight the imperative for biomarker-driven precision medicine and tailored therapeutic strategies.

Safety and tolerability are paramount when extending immunotherapy into the perioperative setting. The addition of ICIs does not increase overall grade 3–5 treatment-related AEs nor compromise surgical timing or feasibility. Our detailed analysis of specific toxicities further refines this safety profile. The spectrum of all-grade treatment-related AEs—most commonly nausea, diarrhea, and decreased neutrophil count—was largely comparable between the ICI–CT and CT-alone groups, indicating that the CT-related toxicity burden was not substantially altered by the incorporation of immunotherapy. Notably, the most frequent immune-related AEs observed with ICI–CT regimens were stomatitis, rash, pruritus, and hypothyroidism, with colitis occurring at a lower frequency. This pattern is consistent with the established toxicity profiles of ICIs and suggests that the predominant irAEs in the perioperative setting are dermatologic and endocrine events, which are generally manageable and non–life-threatening. Nonetheless, grade ≥3 immune-related AEs and serious AEs occur more frequently, necessitating vigilant monitoring and multidisciplinary expertise. Encouragingly, most immune-related AEs are manageable with established immunosuppression protocols without elevating perioperative morbidity or mortality. These data endorse the safe incorporation of ICIs into multimodal curative treatment at centers equipped for early immune-related AE recognition and intervention.

This meta-analysis has several limitations. First and most notably, the number of eligible trials is small (*n* = 4), which constrains the statistical power of the analysis, particularly for subgroup comparisons and safety outcomes where event rates are lower. This is an inherent constraint of the current evidence base, as our systematic search confirmed that these four trials represent the entirety of published phase 2/3 RCTs addressing this specific question as of the search date. Second, there is clinical heterogeneity among the trials regarding CT regimens and types of ICIs, although we employed random-effects models to account for this variability, and no significant statistical heterogeneity was observed for key endpoints like EFS. Third, OS data remain immature, warranting longer follow-up for confirmation. Furthermore, the subgroup analyses presented are exploratory and should be interpreted with caution due to limited statistical power and the potential for type II errors. The findings highlight trends and generate hypotheses for future validation in larger, dedicated studies. Additionally, due to inconsistent reporting across the original trials, we were unable to analyze other critical surgical outcomes such as rates of surgical delay and specific postoperative complications. Future studies should prioritize the standardized collection and reporting of these metrics to fully assess the impact of perioperative immunotherapy on surgical feasibility. Finally, formal assessment of publication bias through funnel plots and Egger’s test is underpowered due to the small number of studies. However, the comprehensive nature of our literature search, which included conference proceedings, and the fact that all major completed trials in this field are represented, reduces the likelihood of significant publication bias affecting our primary conclusions.

Looking forward, priority research areas should focus on addressing key gaps in the current evidence to refine and expand the utility of perioperative chemo-immunotherapy for resectable G/GEJ cancer. This includes expanding biomarker profiling beyond PD-L1 and MSI to incorporate additional predictive and prognostic markers—such as tumor mutational burden, tumor-infiltrating lymphocyte density, and circulating tumor DNA for minimal residual disease detection—which could further optimize patient selection and identify those most likely to derive durable benefit. Additionally, conducting comparative effectiveness trials of different CT backbones, particularly head-to-head evaluations of FLOT versus cisplatin-based doublets, is essential to definitively define the optimal cytotoxic platform for maximizing synergy with ICIs, given the observed differences in efficacy between these regimens in prior trials. Equally important is exploring novel combination strategies—including dual checkpoint blockade or integration of ICIs with anti-angiogenic agents—to overcome inherent or acquired resistance in biologically refractory subgroups, such as patients with diffuse-type tumors or GEJ cancers that showed limited response in the current meta-analysis (25). Finally, evaluating the cost-effectiveness and real-world accessibility of perioperative chemo-immunotherapy across diverse healthcare systems will be critical to supporting equitable implementation, ensuring that this transformative treatment is available to eligible patients regardless of geographic or socioeconomic factors.

## Conclusion

In summary, this meta-analysis of four randomized phase 2/3 trials demonstrates that the addition of immune checkpoint inhibitors to perioperative chemotherapy improves pathological complete response and event-free survival, with an emerging signal of overall survival benefit, while maintaining YHLan acceptable safety profile. However, the evidence remains preliminary given the limited number of available trials, clinical heterogeneity, immature OS data, and underpowered subgroup analyses. These findings support perioperative chemo-immunotherapy as a promising treatment strategy, but confirmation from ongoing phase 3 trials with longer follow-up and standardized reporting will be essential before routine implementation in clinical practice.

## Data Availability

The original contributions presented in the study are included in the article/**Supplementary Material**. Further inquiries can be directed to the corresponding author.
