# Supplementary material for: Addition of immunotherapy to perioperative chemotherapy for resectable gastric and gastroesophageal junction cancer: a meta-analysis of phase 2/3 trials
Source: Front Immunol. 2025 Nov 19;16:1692336. doi: 10.3389/fimmu.2025.1692336 (PMC12672549; doi:10.3389/fimmu.2025.1692336)
Supplement: Supplementary file 1 [file SupplementaryFile1.docx]

**Supplementary Material**

**Addition of immunotherapy to perioperative chemotherapy for resectable gastric and gastroesophageal junction cancer: A meta-analysis of phase 2/3 trials**

**Supplementary Table 1.** Details of search strategy.

**Supplementary Figure 1.** PRISMA flow diagram of study selection.

**Supplementary Figure 2.** Risk of bias graph.

**Supplementary Figure 3.** Risk of bias summary.

**Supplementary Figure 4.** Funnel plot and Egger’s test for pathological complete response.

**Supplementary Table 1.** Details of search strategy.

| **Database** | **Search strategy** |
| --- | --- |
| ***PubMed*** | (((((("Stomach Neoplasms"[Mesh]) OR ("Gastric Cancer")) OR ("Gastroesophageal Junction Adenocarcinoma")) OR ("Gastro-esophageal Junction Cancer")) OR ("Gastro-esophageal Junction Adenocarcinoma")) AND ((((((((((("Immune Checkpoint Inhibitors"[Mesh]) OR ("PD-1 inhibitor")) OR ("PD-L1 inhibitor")) OR (Immunotherapy)) OR (Nivolumab)) OR (Pembrolizumab)) OR (Toripalimab)) OR (Sintilimab)) OR (Atezolizumab)) OR (Durvalumab)) OR (Avelumab))) AND (((Perioperative) OR (Neoadjuvant)) OR (Adjuvant)) Filters: Clinical Trial |


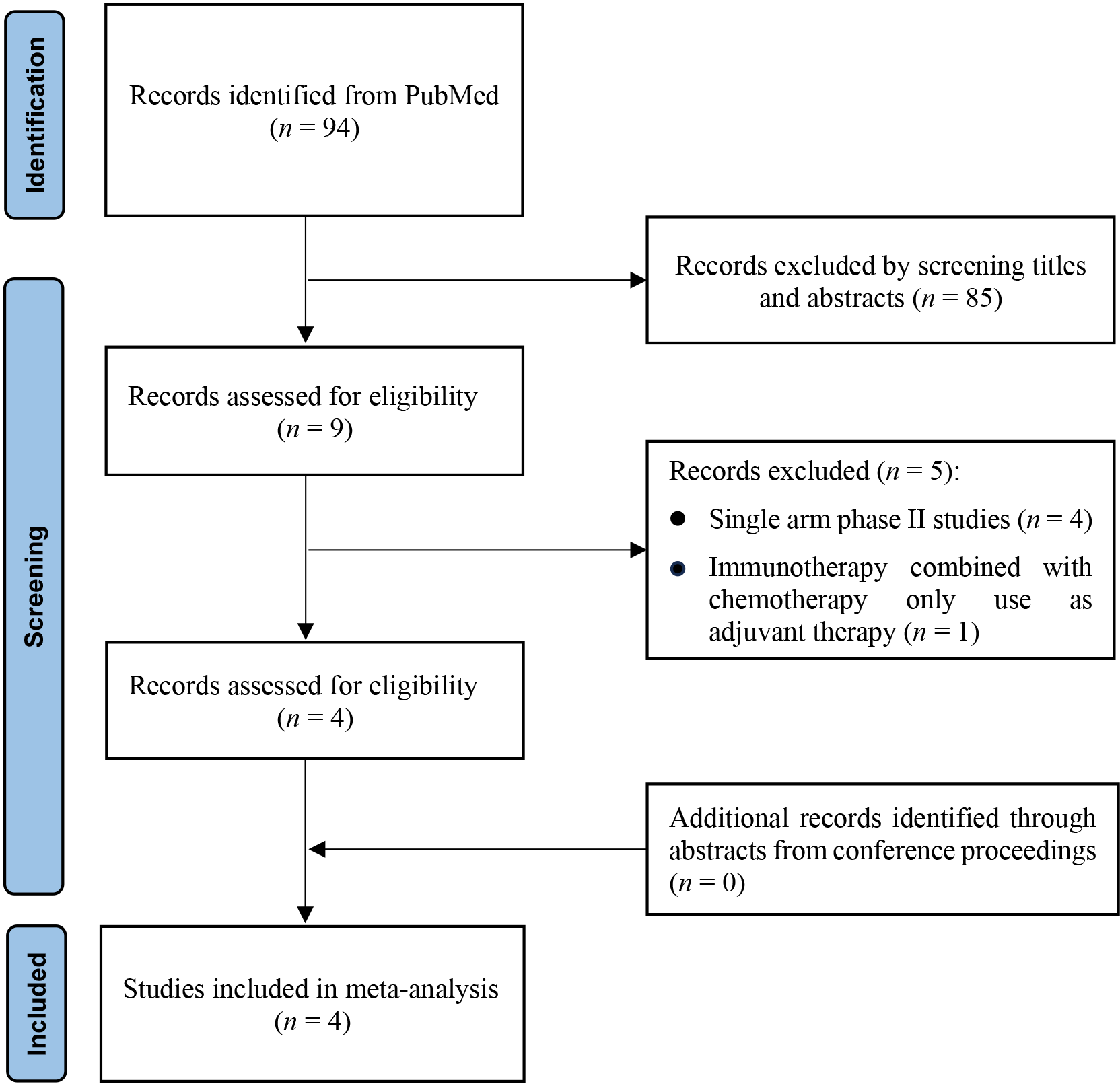


**Supplementary Figure 1.** PRISMA flow diagram of study selection.

**
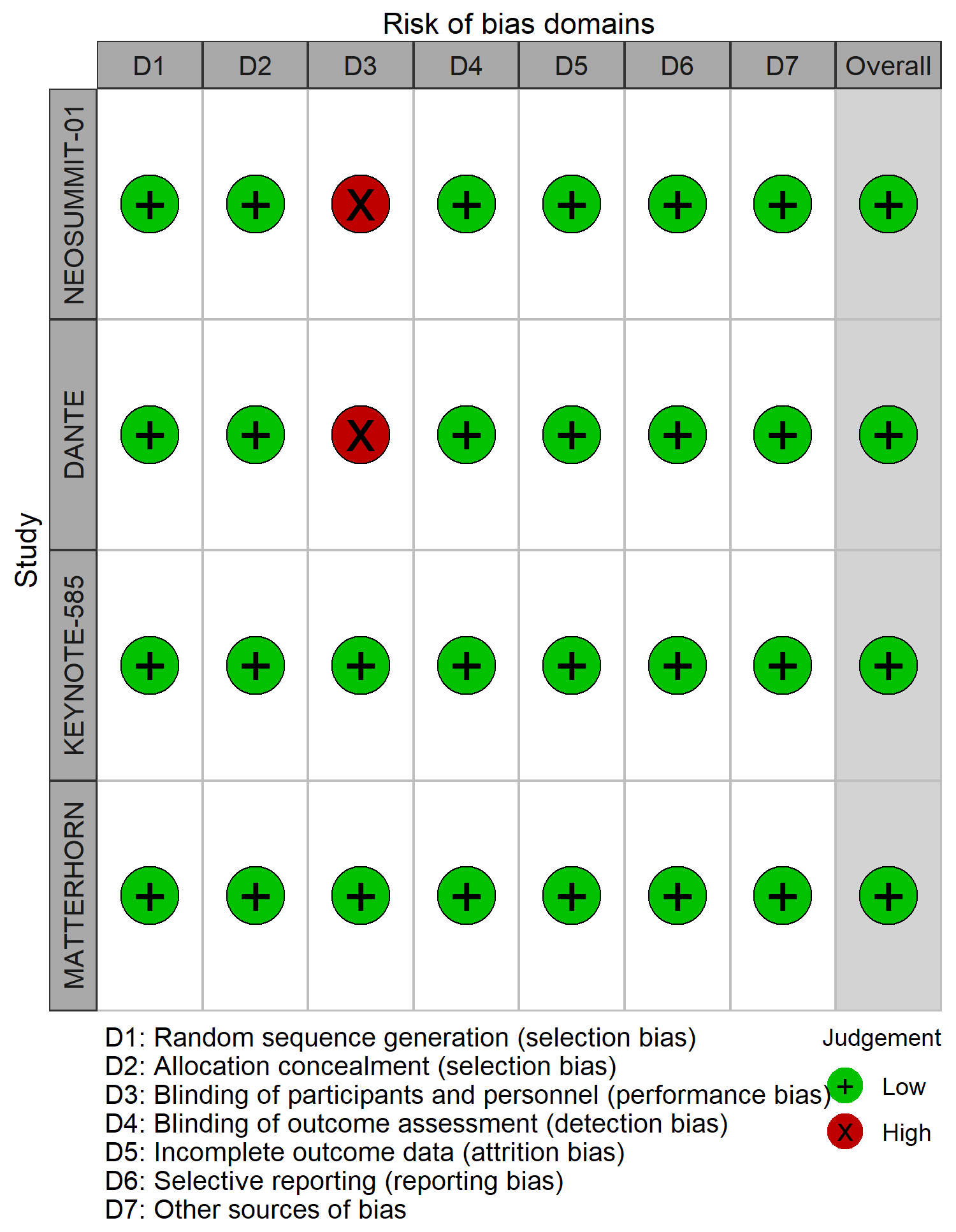
**

**Supplementary Figure 2.** Risk of bias graph.

**
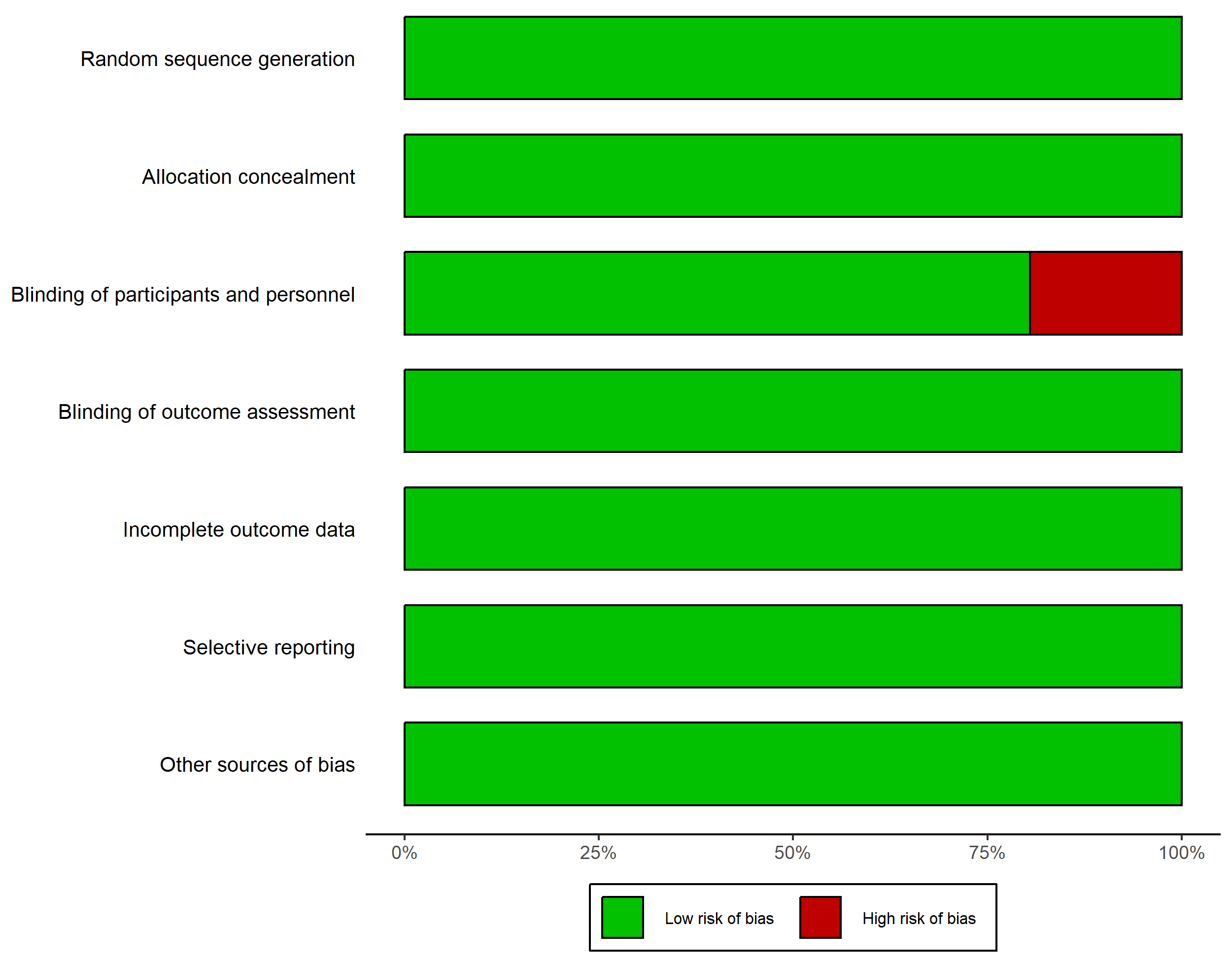
**

**Supplementary Figure 3.** Risk of bias summary.

**
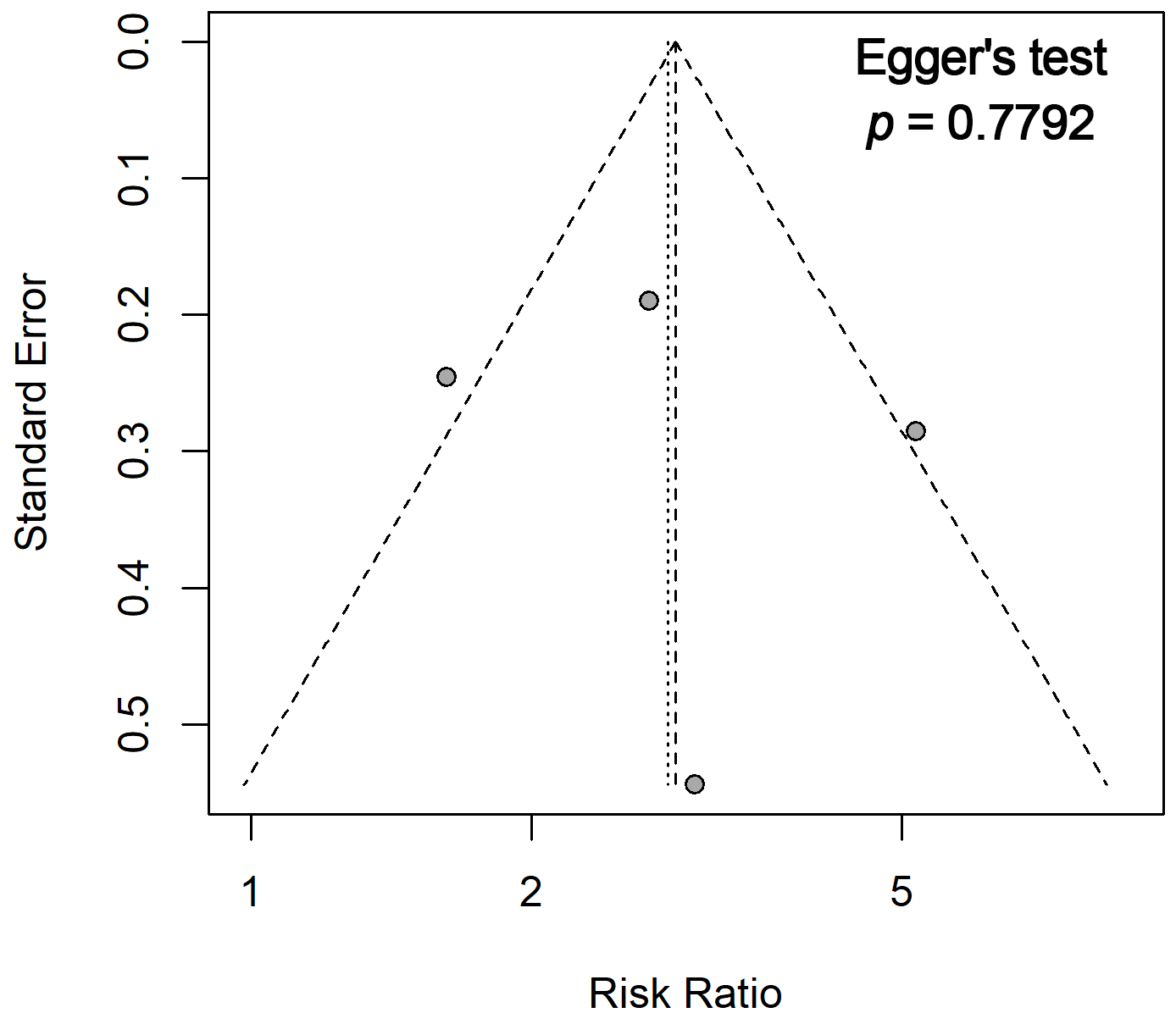
**

**Supplementary Figure 4.** Funnel plot and Egger’s test for pathological complete response.
